# Protein targets of inflammatory serine proteases and cardiovascular disease

**DOI:** 10.1186/1476-9255-7-45

**Published:** 2010-08-30

**Authors:** Ram Sharony, Pey-Jen Yu, Joy Park, Aubrey C Galloway, Paolo Mignatti, Giuseppe Pintucci

**Affiliations:** 1 Department of Cardiothoracic Surgery, Rabin Medical Center and School of Medicine, Tel Aviv University, 39 Jabotinski St., Petah Tikva 49100, Israel; 2 Department of Cardiothoracic Surgery, New York University School of Medicine, 530 First Avenue, New York, NY 10016, USA; 3 Department of Cell Biology, New York University School of Medicine, 530 First Avenue, New York, NY 10016, USA

## Abstract

Serine proteases are a key component of the inflammatory response as they are discharged from activated leukocytes and mast cells or generated through the coagulation cascade. Their enzymatic activity plays a major role in the body's defense mechanisms but it has also an impact on vascular homeostasis and tissue remodeling. Here we focus on the biological role of serine proteases in the context of cardiovascular disease and their mechanism(s) of action in determining specific vascular and tissue phenotypes. Protease-activated receptors (PARs) mediate serine protease effects; however, these proteases also exert a number of biological activities independent of PARs as they target specific protein substrates implicated in vascular remodeling and the development of cardiovascular disease thus controlling their activities. In this review both PAR-dependent and -independent mechanisms of action of serine proteases are discussed for their relevance to vascular homeostasis and structural/functional alterations of the cardiovascular system. The elucidation of these mechanisms will lead to a better understanding of the molecular forces that control vascular and tissue homeostasis and to effective preventative and therapeutic approaches.

## Introduction

Inflammation is a process that delivers defensive tools to injured tissues. Tissue injury implies changes to blood vessels and disruption of normal histological features with rapid recruitment of leukocytes; during this process inflammatory mediators coordinate the response in a manner that preserves both vascular integrity and circulation while allowing extravasation of leukocytes, i.e. their recruitment from circulation to the site of injury. Such perturbation of vascular homeostasis results in biological and biochemical reactions that mediate phenotypic changes both locally and systemically. A typical example of localized phenotypic change is the injury-induced vascular remodeling which ultimately leads to neo-intimal hyperplasia. Systemically, inflammatory perturbation of homeostatic mechanisms affects the vascular tone, often sustaining a hypertensive phenotype.

Activated leukocytes are widely implicated in cardiovascular disease (CVD). Mononuclear cells are recruited to sites of vascular injury thus contributing to foam cells within atherosclerotic plaques [1]; macrophages infiltrate adipose tissue producing a variety of chemokines and cytokines, a key process to the establishment of metabolic syndrome [2]; furthermore, polymorphonuclear cells (PMN) recruited to sites of vascular injury contribute significantly to the development of neo-intimal hyperplasia as they sustain mobilization of medial smooth muscle cells that proliferate and migrate into the neo-intima [3]. Leukocyte activation occurs in all the conditions associated with an increased CVD risk: infection, hypertension, hyperlipidemia, hyperglycemia, obesity, and atherosclerosis [4]. Activated white blood cells discharge into the surrounding milieu reactive oxygen species (ROS) and a variety of proteolytic enzymes, particularly serine proteases [5]. The inflammatory serine protease response is further strengthened by activation of the kallikrein system [6], the involvement of mast cells with the release of chymase and tryptase [7], and activation of the coagulating cascade which ultimately leads to thrombin formation with locally elevated levels of thrombin activity [8,9].

Sequencing of the human genome shows that more than 2% of all human genes are proteases or protease inhibitors, indicating the overall importance of proteolysis in human biology [10]. The human degradome consists of at least 561 proteases and homologs, which are distributed into 186 metallo-, 178 serine-, 21 aspartic-, 148 cysteine-, and 28 threonine- proteases [11]. A number of studies have emphasized that in addition to their direct proteolytic effect(s) proteases possess a variety of regulatory functions that are mediated through intracellular signaling pathways, caspase-like enzyme activity and/or regulation of specific cytokines and signaling receptors. Therefore, proteases are now considered as multifunctional, hormone-like signaling molecules that play a pivotal role in various physiological and pathological processes [12]. Protease-mediated signaling can proceed via specific protease-activated receptors (PAR) and/or PAR-independent mechanisms.

In this review we will focus on serine proteases, which have a direct effect on degradation of proteins of the extracellular matrix including collagen, elastin, and fibronectin [13]. Pro-inflammatory effects of serine-proteases will be discussed particularly in light of their relevance to CVD. We will also consider serine proteases' specific targets whose induction and/or degradation has a demonstrated impact on their biological activity and the pathogenesis of cardiovascular disease.

### Protease-activated receptors (PARs)

Most serine proteases transduce their signal(s) into the cell by interacting with specific cell membrane receptors. This mechanism controls a number of relevant cellular effects of serine proteases. Protease-activated receptors (PARs) are a unique class of transmembrane G protein-coupled receptors (GPCRs) that play a critical role in thrombosis, inflammation, and vascular biology. Leger et al. [14] reported that all the four PARs described to date are expressed in various types of cells present in the vasculature and modulate the responses to coagulation proteases during thrombosis and inflammatory states. PAR_1 _and PAR_2 _expressed in smooth muscle cells and PAR_1_, PAR_2_, and PAR_4 _expressed in macrophages activate inflammatory and proliferative pathways in atherosclerotic lesions [15]. Rodent platelets lack PAR_1 _and instead use PAR_3 _to enhance thrombin cleavage of the lower-affinity PAR_4 _[14]. PAR_2 _is mostly a receptor for the tissue factor/factor VIIa/factor Xa complex and is also a preferred target of trypsin, but not thrombin [16,17]. PAR_1 _and PAR_4 _signaling show considerably different kinetics and indeed appear to have distinct functions in platelet aggregation [14]. PAR_1 _is a high-affinity receptor for thrombin by virtue of a hirudin-like sequence that resides in its N-terminal extracellular domain [18,19]. Signal transduction via PAR_1 _is fast and transient, and is followed by a prolonged signaling arising from PAR_4_, a receptor normally more slowly activated by thrombin. For activation, PAR_1 _exo-domain harbors a hirudin-like sequence element that interacts with thrombin. PAR_4 _has an optimal cleavage sequence that provides high-affinity interactions with the active site and uses an anionic cluster for slow dissociation from the cationic thrombin. PAR_1 _also acts as a cofactor for thrombin activation of PAR_4 _which provides a mechanistic basis to understand PAR_1_/PAR_4 _synergy [14]. Human platelets express both PAR_1 _and PAR_4 _which give rise to a coordinated thrombin response and subsequent activation of the glycoprotein GP IIb/IIIa, a fibrinogen receptor, with formation of platelet-rich thrombi.

PARs have a unique mechanism of activation that distinguishes them from other seven transmembrane GPCRs that are activated reversibly by small hydrophilic molecules to elicit cellular responses [20]. PAR activation involves the proteolytic unmasking of the receptor's N- terminus to reveal a cryptic tethered ligand (TL) that binds to and activates the receptor [21]. PARs, with the exception of PAR_3_, are also activated by short synthetic peptide sequences derived from the sequences of the proteolytically revealed TL [19,22]. It is worth mentioning that proteases can also exert a negative regulation through PARs by 'disarming' the receptor by cleavage at a non-receptor activating site which results in removing the TL. Minami and collaborators [23] have described several steps following initial stimulation of PAR. Thrombin activates PAR_1 _by binding to a unique site in the extracellular domain of the receptor, resulting in cleavage between Arg41 and Ser42 and subsequent exposure of a new N-terminus. The unmasked tethered ligand (SFLLRN) interacts with the extracellular loop 2 of the receptor (amino acids 248 to 268), resulting in receptor activation [24]. Once cleaved, PAR_1 _transmits the signal across the plasma membrane to intracellular G proteins. The G proteins are in turn associated to a number of signal intermediates that include mitogen-activated protein kinases (MAPKs), protein kinase C (PKC), phosphatidyl-inositol 3-kinase (PI3-K), and Akt. In normal platelets, this process culminates in morphologic changes of the cells, platelet-platelet aggregates, control of release of platelet dense granules and a rapid rise in intracellular calcium [25-27]. Thrombin signaling results in changes in downstream transcription of genes involved in cell proliferation, inflammation, leukocyte adhesion, vasomotor tone, and hemostasis. In addition, thrombin controls post-transcriptional changes such as calcium influx, cytoskeletal reorganization, and release of soluble mediators and growth factors into the extracellular matrix [23].

In reviewing the role of PARs expressed in the vascular endothelium Leger et al. [14] emphasized that their activation mediates responses involved in contractility, inflammation, proliferation, and repair complementing the functions of platelet PAR_1 _by localizing the thrombus to the site of vascular injury. This process involves calcium mobilization and secretion of Weibel-Palade bodies, which harbor vWF multimers and the P-selectin adhesion molecule [28]. Activated PAR_1 _thus mediates the inflammatory process in the endothelium, causes cytoskeletal rearrangements and induces cell contraction and rounding [29,30]; this mechanism destabilizes cell-cell contacts with subsequent increase in vascular permeability which facilitates the passage of molecules and cells from the blood into sub-endothelial compartments while tissue factor (TF) and collagen are exposed to the vascular bed. Recent studies have indicated that PAR activation by thrombin, factor Xa, and activated protein C (APC) can either promote or protect against changes in vascular permeability depending on the status of the endothelium [31,32]. PAR_1 _signaling can also play opposing roles in sepsis, either promoting coagulation and inflammation or reducing sepsis lethality due to APC therapy. Recombinant human activated protein C (hrAPC) was developed to reduce excessive coagulant and inflammatory activity during sepsis. Basic and clinical research studies have suggested that these pathways contribute to the pathogenesis of this lethal syndrome and are inhibited by rhAPC. Recent data showed that treatment with hrAPC in septic patients may improve muscle oxygenation and reperfusion and, furthermore, hrAPC treatment may increase tissue metabolism [33]. Similar to thrombin, which is a serine protease of the coagulation cascade that induces inflammatory responses and controls endothelial barrier permeability, APC, an anti-coagulant protease, also activates PAR_1_. Unlike thrombin, however, APC elicits anti-inflammatory responses and protects against endothelial barrier dysfunction induced by thrombin. Thus, a mechanism of protease-selective signaling by PAR_1 _has been suggested, called the PAR_1 _paradox. Russo et al. [34] have recently reported that thrombin and APC signaling were lost in PAR_1 _deficient endothelial cells, indicating that PAR_1 _is a major effector of protease signaling. They reported that thrombin caused robust Rho-A signaling but not Rac-1 activation, whereas APC stimulated a marked increase in Rac-1 activation but not Rho-A signaling, consistent with the opposing functions of these proteases on endothelial barrier integrity. Using cells lacking caveolin-1, an endothelial cell membrane protein involved in receptor-independent endocytosis, these Authors also found that APC selective signaling and endothelial barrier protective effects were mediated through compartmentalization of PAR_1 _in caveolae by a novel mechanism of PAR_1 _signal transduction regulation. Acute blockade of the APC pathway with a potent inhibitory antibody revealed that thrombin/PAR_1 _signaling increases inflammation-induced vascular hyper-permeability. Conversely, APC/PAR_1 _signaling and the endothelial cell protein C receptor (EPCR) prevented vascular leakage, and pharmacologic or genetic blockade of this pathway sensitized mice to LPS-induced lethality. Hence, PARs may play a role in disease states characterized by decreased barrier function, including sepsis and systemic inflammatory response syndrome, and their pharmacological modulation may therefore ameliorate serious clinical states.

Vascular smooth muscle cells (VSMC) represent sites of PAR_1 _over-expression in human atherosclerotic arteries, including regions of intimal thickening [35]. *In vitro *studies revealed that activation of PAR_1 _triggers mitogenic responses in VSMC and fibroblasts [36]. Moreover, a PAR_1 _neutralizing antibody reduced intimal hyperplasia in a catheter-induced injury model of restenosis [37]. Finally, studies using PAR_1 _deficient mice and small molecule PAR_1 _antagonists further implicated PAR_1 _in thrombosis and restenosis [38].

There are a number of evidences that the effect of PARs on local vessel vaso-reactivity may vary greatly depending on whether the endothelium is healthy or rather in the context of an atherosclerotic lesion. Stimulation of intact coronary arteries with thrombin or PAR_1 _agonist peptides elicited relaxation [39] while in atherosclerotic human coronary arteries stimulation of PAR_1 _failed to elicit relaxation and in some cases caused marked contraction [40].

As PARs and other GPCRs belong to the group of transmembrane receptors, they modulate G protein signaling on the inside surface of the receptor [25]. Several studies utilized lipidated peptides based on the intracellular loop sequences of the GPCRs of interest like pepducins, which bind to the receptor-G protein interface on the inner leaflet of the plasma membrane. These molecules have been studied extensively in the context of PAR_1 _and PAR_4 _signaling in platelets and in animal models of thrombosis, inflammation, angiogenesis, and migration and invasion of cancer cells [14,41-43]. PAR_1 _and PAR_2 _mediate various vascular effects including regulation of vascular tone, proliferation and hypertrophy of smooth muscle cells and angiogenesis. Since proteases are activated under pathological conditions such as hemorrhage, tissue damage, and inflammation, PARs are suggested to play a critical role in the development of functional and structural abnormality in the vascular lesion [44,45]. Therefore, development of new strategies for the prevention and therapy of vascular diseases can be achieved by understanding the functional role of PARs in the vascular system.

### PAR-independent serine protease activity affecting the cardiovascular system

Although many studies have explored the role of PARs in protease signaling a number of alternative mechanisms can account for protease biological activity in controlling the cardiovascular system. Here we will discuss inflammatory serine proteases as they exert their biological activity on the cardiovascular system by targeting cytokines, growth factors, membrane receptors, and other vasoactive proteins with or without the involvement of PARs (Table 1).

**Table 1:** Summary of protein targets of inflammatory serine proteases.

Serine protease	Targets	Function	Role in cardiovascular disease	References
Elastase	E-cadherin, GM-CSF, IL-1, IL-2, IL-6, IL8, p38^MAPK^, TNFα, VE-cadherin	Degrades ECM components Regulates inflammatory response Activates pro-apoptotic signaling	Promotes atheromatous plaque formation Promotes vascular damage Promotes ischemia and reperfusion injury Triggers endothelial cell apoptosis	[13,46-50,57,78,89,90,92]

Cathepsin G	EGF, ENA-78, IL-8, MCP-1, MMP-2, MT1-MMP, PAI-1, RANTES, TGFβ, TNFα	Degrades ECM components Chemo-attractant of leukocytes Regulates inflammatory response Promotes apoptosis	Initiates calcification and fibrosis of aortic valve Promotes tissue remodeling Induces VSMC proliferation Modulates coagulation	[58-65,67,70,78,88]

PR-3	ENA-78, IL-8, IL-18, JNK, p38^MAPK^, TNFα	Promotes inflammatory response Activates pro-apoptotic signaling	Promotes vascular damage Triggers endothelial cell apoptosis	[78,85,89,90]

Thrombin	FGF-2, HB-EGF, Osteo-pontin, PDGF, VEGF	Modulates activity of vascular growth factors, chemokines and extracellular proteins Strengthens VEGF-induced proliferation Induces cell migration Angiogenic factor Regulates haemostasis	Promotes angiogenesis and vascular remodeling Promotes coagulation and platelet aggregation	[14,23,31,98-101,118,122,123,173,174]

Kallikreins	high molecular weight kininogen, pro-urokinase	Modulate relaxation response Contribute to inflammatory response Fibrin degradation	Impact fibrinolysis and vascular tone Induce relaxation of contracted aortas	[132-136,140,141]

Tryptase and Chymase	angiotensin I, fibrinogen, pro-urokinase, TGFβ	Activate pro-urokinase Promote angiogenesis Modulate coagulation cascade	Affect blood pressure Promote angiogenesis Promote remodeling of fibrotic tissue Impact fibrinolysis and vascular tone	[7,142-147]

#### Neutrophil serine proteases

Elastase is the major serine protease contained in the azurophile granules of polymorphonuclear cells (PMN, or neutrophils). When discharged upon PMN activation neutrophil elastase has a direct effect on the degradation of extracellular matrix components, including collagen, elastin and fibronectin [13]. In addition, elastase has a pro-inflammatory effect. It degrades inter-endothelial VE-cadherin and inter-epithelial E-cadherin, promoting permeability through these cell layers [46,47]. Elastase also stimulates the secretion of granulocyte-macrophage colony-stimulating factor (GM-CSF), IL-6 and IL-8 from epithelial cells [48,49] and at the same time is capable of degrading the cytokines IL-1β, IL-1, IL-8, IL-2 [50]. This effect further enhances leukocyte migration and propagates inflammation.

The net effect of proteolytic activity depends on the balance between a pro-inflammatory and an anti-inflammatory state. In various diseases an imbalance in the ratio of proteases and their physiological inhibitors has a role in the progression of the pathologic process. For example, inherited deficiency of 1-proteinase inhibitor ( 1-PI), the principal extracellular inhibitor of neutrophil elastase, increases the risk of severe early-onset emphysema [51]. Conversely, Ortiz-Muñoz et al. [52] have recently shown the presence of _1_-antitrypsin in high-density lipoprotein (HDL) that possesses a potent anti-elastase activity. The same Authors also reported that HDL-associated _1_-antitrypsin was able to inhibit extracellular matrix degradation, cell detachment, and apoptosis induced by neutrophil elastase in human VSMCs and in mammary artery cultured *ex vivo *[52].

Thrombus formation at the surface of an atherosclerotic plaque in coronary or carotid arteries may cause acute occlusion and subsequent complications such as myocardial infarction or stroke leading to serious clinical conditions. Such pathological events caused by rupture of a thin-capped fibro-atheroma containing a lipid-rich necrotic core lead to the exposure of plaque-associated tissue factor to circulating coagulation factors, platelet activation, and subsequently to the formation of an occlusive thrombus [53,54]. The atherosclerotic plaque may be stable for a long time or rather prone ("vulnerable") to disruption. A huge scientific effort is justifiably under way in order to characterize the vulnerability of the atherosclerotic plaque.

Extracellular protease levels increase with the major coronary risk factors. Smoking and Type 1 diabetes increase plasma elastase levels [55,56]. Dollery et al. [57] showed in human tissue that fibrous and atheromatous plaques but not normal arteries contained significant amounts of neutrophil elastase. Moreover, elastase abounded in the macrophage-rich shoulders of atheromatous plaques with histological features of vulnerability. These Authors also showed by *in situ *hybridization that elastase was highly expressed in macrophage-rich areas, indicating local production of this enzyme.

Cathepsin G is another serine protease of PMN azurophile granules that hydrolyses several types of proteins. Cathepsin G exerts potent pro-inflammatory properties [58]. It plays a role in the degradation of extracellular matrix components and cytokines and also as a chemo-attractant for leukocytes, including T cells. Cathepsin G has a potent elastolytic activity and thus plays a key role in tissue remodeling [59,60]. Cathepsin G cleaves and activates G protein-coupled receptors (GPCRs) as a mechanism to modulate coagulation and tissue remodeling at sites of injury and inflammation (see above). Cathepsin G also induces activation of the matrix metallo-proteinases MMP-2 and MT1-MMP. In fact, myocytes treated with an MMP-2 inhibitor display reduced ERK-1/2 phosphorylation and attenuated apoptosis induced by cathepsin G. In addition, inhibition of MT1-MMP by either TIMP-2 or neutralizing MT1-MMP antibodies blocks cathepsin G-induced MMP-2 activation and ERK-1/2 phosphorylation [61]. A decrease in apoptosis has also been observed in a model of cathepsin G^-/- ^mice [62]. Cathepsin G-mediated cell detachment and apoptosis have also been demonstrated in cultured cardiomyocytes [63]. Our group and others have shown the role of MT1-MMP in cathepsin G-induced MMP-2 cleavage and epidermal growth factor receptor (EGFR) trans-activation [61,64]. Cathepsin G-induced cardiomyocyte apoptosis involves an increase in EGFR-dependent activation of protein tyrosine phosphatase SHP2 (Src homology domain 2-containing tyrosine phosphatase 2) which promotes focal adhesion kinase dephosphorylation and subsequent cardiomyocyte anoikis, the apoptotic response of cells to the absence of cell-matrix interactions [64].

The chemokine RANTES (Regulated upon Activation, Normal T-cell Expressed and Secreted) is strongly induced by viral and bacterial infections and plays a role in allergic diseases, asthma exacerbation, interstitial pneumonia, allograft rejection and in some types of cancers. Recently, RANTES has been shown to induce VSMC proliferation in an animal model of graft arterial disease [65]. Interestingly, the RANTES specific allele is associated with the presence and severity of coronary artery disease [66]. Cathepsin G mediates the regulation of RANTES signaling pathway. In fact, Lim et al. [67] showed that cathepsin G promotes post-translational processing of RANTES into a variant lacking N-terminal residues, called 4-68 RANTES, which exhibits less efficient binding to the chemokine receptor CCR5 and lower chemotactic activity [68]. In this study, it was also shown that the cathepsin G inhibitor Eglin C abrogated cell-mediated production of 4-68 RANTES. Furthermore, neutralizing cathepsin G antibodies also abrogated RANTES digestion in neutrophil cultures. These findings demonstrate that cathepsin G proteolytic activity operates a tight control of RANTES.

Cathepsin G enzymatic activity can lead to generation of angiotensin II which in turn induces the expression of monocyte chemoattractant protein-1 (MCP-1) [69], and also triggers an angiotensin II-dependent profibrotic response mediated by transforming growth factor-beta 1 (TGF-β1). In addition to its pro-fibrotic effect, cathepsin G-mediated TGF-β1 formation also initiates calcification of the aortic valve [70]. Hence, cathepsin G-mediated TGF-β1 formation may be associated with both fibrosis and calcification of the aortic valve, two important mechanisms of valvular disease. It has also been reported that cathepsin G expression is significantly increased in human stenotic aortic valve and that this is associated with the formation of atheroma of the carotid artery [71].

Reperfusion of ischemic tissues induces an inflammatory response [72,73]. This process is associated with cytokine and chemokine production and expression of adhesion molecules, neutrophil infiltration and subsequent tissue damage [74,75]. Studies using animal models have shown that neutrophil depletion before reperfusion or blockade of neutrophil infiltration into the ischemic tissue results in attenuating the injury associated with ischemia-reperfusion [76,77]. Cathepsin G^-/- ^mice have normal development of neutrophils but an abnormal wound healing response. These mice also present a reduced tissue injury in a model of renal ischemia-reperfusion; cathepsin G thus appears to be a critical factor for sustaining neutrophil-mediated acute tissue pathology and subsequent fibrosis [62].

Tumor necrosis factor-alpha (TNF-α) is one of the major cytokines involved in the inflammatory response. Proteolytic cleavage of the membrane-bound pro-form of TNF-α is a requirement for its biological activity. Elastase and cathepsin G are both involved in the shedding of membrane-bound TNF-α. Elastase and Proteinase-3 (PR-3), another protease contained in the azurophile granules, are both able to process TNF-α *in vitro *into its soluble, active form [78] whereas serine protease inhibitors suppress the secretion of TNF-α from activated macrophages [79,80].

The ADAM (A Disintegrin And Metallo-proteinase) family of peptidases are involved in a process called 'shedding', i.e the cleavage and release of a soluble ectodomain from membrane-bound pro-proteins. ADAM metallopeptidase with thrombospondin type 1 motif, 17 (ADAMTS17) is the main physiological TNF-α processing enzyme. However, studies conducted with cultured fibroblasts isolated from ADAMTS17^-/- ^animals have shown that both elastase and cathepsin G release soluble TNF-α (diminishing the levels of the membrane-bound form and increasing the levels of biologically active, soluble TNF-α). In contrast to cathepsin G, elastase is also able to further degrade, and thus inactivate, soluble TNF-α, although at higher concentrations [81,82]. Thus, these proteases modulate the levels of soluble TNF-α contributing to control its pro-inflammatory activity in the tissue.

The cytokine IL-18 plays a role in inflammatory states like sepsis, arthritis and inflammatory bowel disease [83,84]. Its inactive precursor pro-IL-18 is generally cleaved by caspase-1 to its active form. *In vitro*, PR-3 activates IL-18 independently of caspase-1 activity. This has been elegantly confirmed using a caspase-1-deficient mouse model [85].

In addition to the cytokines mentioned above neutrophil serine proteases can cleave a number of ligand-binding cytokine receptor ectodomains. This cleavage appears to modulate the cellular response by inactivating the receptor or prolonging the cytokine half-life [86,87] with subsequent down-regulation of the inflammatory response. It should be noticed that proteolytic cleavage of cytokines like IL-8 and ENA-78 by PR-3 and cathepsin G leads to a more active form of the chemokines. Therefore, neutrophil serine proteases, by activating specific receptors and releasing or inactivating a number of cytokines, modulate the dynamic state of cytokines at the site of inflammation.

The potential effects of cathepsin G in cardiovascular disease may also derive from its control of the fibrinolytic system. While investigating cathepsin G biological effects on human endothelial cells we discovered that cathepsin G induced suppression of tissue-type plasminogen activator (tPA) activity and that this effect was mediated by release of its physiological inhibitor plasminogen activator inhibitor 1 (PAI-1) from the endothelial extracellular matrix [88]. Interestingly, in the same study we discovered that cathepsin G was able to release PAI-1 from platelets, thus strengthening its potential role as a thrombogenic factor.

The complexity of the impact of leukocyte-derived proteases processing of specific substrates on intracellular signaling pathways has several repercussions on the vascular cell phenotype. Yang et al. [89] found that release of PR-3 and elastase by activated neutrophils during acute inflammation may result in vascular damage by triggering endothelial cell apoptosis. This group also reported that the release of neutrophil and monocyte proteases, such as PR-3 and elastase, can facilitate the passage of these white blood cells through the endothelial cell layer with the concomitant activation of pro-apoptotic signaling pathways such as the stress-activated mitogen-activated protein kinases (MAPKs). Accordingly, inhibition of the MAPK JNK blocked PR-3-induced apoptosis, and also inhibition of p38^MAPK ^blocked PR3- and elastase-induced apoptosis, indicating that these pathways are required for activation of apoptosis by these proteases [90].

The inhibition of neutrophil elastase ameliorates ischemia and reperfusion injury as shown in a mouse liver model. Treatment with an elastase inhibitor decreased local neutrophil infiltration and diminished apoptosis as determined by terminal deoxy-nucleotidyl transferase-mediated dUTP nick-end labeling (TUNEL) staining and caspase-3 cleavage. Thus, targeting neutrophil elastase represents a useful approach for preventing ischemia and reperfusion injury [91] which suggests potential applications for the therapy of cardiovascular diseases. In addition, an *in vitro *study using isolated PMN from venous blood of healthy volunteers showed that also C-reactive protein (CRP) degradation products generated by NE promoted neutrophil apoptosis and cell death. Therefore, cleavage of CRP by neutrophil elastase may have a role in modulation of inflammatory injury (see below for more data on CRP) [92].

However, it should be pointed out that studies on different cell types, i.e. epithelial cells, have shown that there is a dual cellular response to elastase in acute inflammation that includes the activation of both pro-apoptotic and pro-survival pathways, the balance of which ultimately determines the cell's fate [93].

#### Thrombin and growth factor signaling

The cardiovascular system's development and maintenance are tightly controlled by the concerted activities of a variety of vascular growth factors. These include vascular endothelial growth factor (VEGF), fibroblast growth factors (FGFs), heparin-binding epidermal growth factor (HB-EGF) and platelet-derived growth factors (PDGFs) which all act via specific cell membrane receptors known as receptor tyrosine kinases (RTKs); their activation triggers multiple intracellular signaling pathways [94]. The impact of these growth factors on angiogenesis and vascular remodeling has been widely documented and several approaches aimed to interfering with these processes by selectively inhibiting the growth factor activity directly or indirectly have shown a variable degree of success [95-97]. Interestingly, thrombin, a serine protease activated upon tissue injury and inflammation, modulates the activity of most vascular growth factors, which in part explains its angiogenic properties. In fact, thrombin not only releases VEGF from tumor cells but also strengthens VEGF-induced proliferation of vascular endothelial cells via upregulation of VEGF receptors expression [98]. The thrombin-induced shedding of HB-EG from the cell membrane controls VSMC proliferation by transactivating the EGF receptor [99]; interestingly, the intracellular response triggered by thrombin via this mechanism induces a biphasic activation of the ERK-1/2 pathway which seems to be mediated by MMPs [100]. Thrombin also modulates the expression of PDGF, a growth factor implicated in the stabilization of angiogenic vessels through pericyte maintenance as well as in the development and progression of intimal hyperplasia, a process synergistically supported in concert with basic FGF (FGF-2) [101,102]. Thrombin strengthens the biological activity of FGF-2 which mediates its effects, particularly on VSMC [103-109]. Our group has characterized several effects of FGF-2 on vascular cells, including its critical role in inducing endothelial cell migration via activation of the MAPKs ERK-1/2 [110,111]. As our recent findings have unraveled a novel mode of interaction between thrombin and FGF-2 (see below) we will discuss these two molecules in further detail.

FGF-2 is the prototypic member of a family of small proteins with pleiotropic effects [112]. The *fgf-2 *human gene is expressed in different molecular weight forms generated by alternative translation from a single mRNA transcript. Translation of the 18 kDa form or low molecular weight (LMW) FGF-2 is initiated from a classical AUG codon, whereas the 22, 22.5 and 24 kDa forms, collectively known as high molecular weight (HMW) FGF-2, are translated from alternative CUG codons upstream of the AUG [113,114]. The HMW forms of FGF-2 are therefore colinear extensions of 18 kDa FGF-2 [115,116]. Within the cell HMW FGF-2 is predominately localized to nuclei and nucleoli, whereas LMW FGF-2 is mostly cytoplasmic; importantly, both forms are also detected into the extracellular environment although the mechanism of their release remains poorly understood. Selective expression of HMW FGF-2 is induced by stress conditions such as heat shock and oxidative stress, and by specific cytokines and growth factors (reviewed in Yu *et al*. [116]). The different FGF-2 forms have been implicated in various pathological processes, including vascular remodeling and arterial restenosis, neuronal regeneration after injury and tumor growth [116,117].

The serine protease thrombin is a key regulator of vascular integrity and homeostasis; it is a key enzyme of the coagulation cascade, angiogenic factor, inflammatory mediator, platelet agonist and regulator of vascular cell functions [118]. Generation of thrombin through activation of the tissue factor-dependent coagulation cascade is also well described in malignancy [119]. Accordingly, thrombin has been shown to promote tumor growth and metastasis, an effect in part attributed to its angiogenic activity [120,121] and its induction of chemokines, growth factors and extracellular proteins [122,123]. Most of thrombin effects are mediated by activation of specific protease-activated receptors (PARs) and their downstream intracellular signaling [124] (see above).

**Figure 1:**
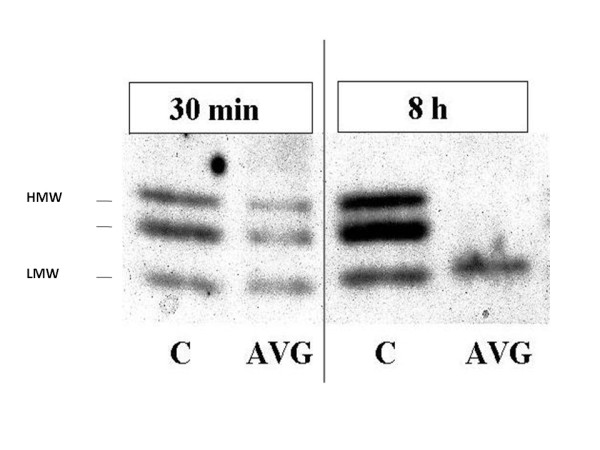
**Western blotting analysis of FGF-2 expression in canine arterialized vein grafts (AVG) or in control femoral veins (C) harvested at the indicated times after grafting showed disappearance of HMW FGF-2 at 8 h and the presence of a band in the 18 kDa range**.

**Figure 2:**
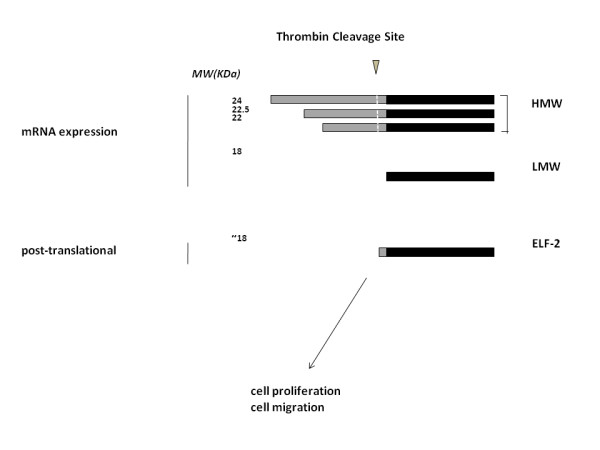
**Schematic of the different human FGF-2 forms: high molecular weight (HMW, 24, 22.5, 22 kDA) or low molecular weight (LMW, 18 kDa) expressed upon alternative translation from a unique mRNA**. The HMW forms (black plus grey bars) are colinear extensions of LMW FGF-2 (black bar only), and are inactive or inhibitory on vascular cell migration. Upon tissue injury and cellular damage, thrombin cleaves all HMW FGF-2 forms exported in the extracellular environment in at least three different bonds (indicated as a unique white dotted line) upstream of the initiating methionine of 18 kDa FGF-2 thus generating an eighteen kDa-like FGF-2 form (ELF-2) that induces vascular cell proliferation and migration.

Thrombin effects on vascular cells have been demonstrated to depend on FGF-2; FGF-2 is also released by thrombin from the heparan sulfates that act as molecular storage for its bioavailability in the extracellular matrix [103,106,107,109,125]. While investigating the vascular response of a vein utilized as bypass graft in the arterial circulation (vein graft arterialization), we observed a dramatic and rapid increase in vein graft-associated thrombin activity [9]. Because this finding was paralleled by an apparent increase in LMW FGF-2 and disappearance of HMW FGF-2 in the vein graft (Fig. 1), we investigated the effect of thrombin on FGF-2 expression in various vascular cell cultures. Adding thrombin to the culture medium resulted in loss of cell-associated HMW FGF-2 and concomitant accumulation of LMW FGF-2. The rapid kinetics of this effect (5 min) suggested that it was not the result of a translational control by thrombin. In fact, using FGF-2^-/- ^mouse endothelial cells engineered to express solely the human HMW FGF-2 we demonstrated that thrombin cleaves HMW FGF-2 into an 18 kDa-like form of FGF-2 (ELF-2). Importantly, we showed that cleavage of HMW FGF-2 into ELF-2 mediates thrombin mitogenic and pro-migratory effects on endothelial cells independent of PAR activation [126] (Fig. 2). Thrombin cleavage of HMW FGF-2 thus represents a rapid mechanism of post-translational control of FGF-2 activity. Therefore, one must conclude that thrombin induces FGF-2 activity by both translation-dependent and -independent mechanisms. Both LMW and HMW FGF-2 are massively exported from the cell upon cell death, injury or sub-lethal damage (reviewed in Yu *et al*. [116]). Accordingly, release of all forms of FGF-2 occurs significantly in injured endothelial cells [126]. *In vivo *vascular injury is accompanied by localized endothelial and smooth muscle cell damage as well as thrombin generation. These conditions are therefore ideal for thrombin-HMW FGF-2 interactions in the extracellular environment.

#### The kallikrein-related peptidases

Kallikrein-related peptidases constitute a family of 15 (chymo)-trypsin-like proteases (KLK 1-15) that are secreted as inactive zymogens and can exhibit either trypsin- or chymotrypsin-like activity upon activation [127-129]. Kallikreins are abundantly expressed in many tissues as well as in circulation and are upregulated in disease. Increased levels of human kallikreins have been detected in ovarian and breast cancer patients [130,131] as well as at sites of inflammation [132,133]. Human kallikrein 14 (KLK 14) is the main physiological regulator of PAR_2 _in many settings, in addition to other serine proteases like trypsin, factor VIIa/Xa, and tryptase. Oikonomopoulou et al. [134,135] comprehensively described the role of tissue kallikreins on PARs. Based on the fact that activation of either PAR_1 _or PAR_2_, but not PAR_4_, induces an endothelium-dependent nitric oxide-mediated aorta relaxation [136,137] an aorta ring relaxation assay using rat or mouse vascular tissue was performed to determine whether human kallikreins could activate PARs in intact tissues. These Authors found that human KLK 5, 6, and 14 caused relaxation of rat aorta that had been pre-constricted with phenylephrine. Human KLK 14 ability to induce relaxation of pre-contracted aortas was further investigated using wild-type vs PAR_2_-null mice. In endothelium-intact murine aorta preparations human KLK 14 caused a relaxation response that was comparable to that of acetylcholine. Conversely, KLK 14 failed to cause relaxation in pre-constricted tissues obtained from PAR_2_-null mice in which the relaxation response could still be observed in the presence of PAR_1 _activators (TFLLR-NH_2 _or thrombin). Thus, signaling via human, rat, and murine PARs can be regulated by kallikreins. Interestingly, kallikreins, and specifically human KLK 14, contribute to the inflammatory response [137-139].

As in the case of other serine proteases kallikreins can cleave a number of protein targets of interest for the cardiovascular system. One major example of such a mechanism is certainly the cleavage of high molecular weight kininogen operated by both plasma and tissue kallikreins off the platelet surface [140]. As the cleavage of membrane-bound high molecular weight kininogen by kallikrein releases the potent vasodilator bradykinin this mechanism has an obvious impact on vascular tone.

Another interesting target of plasma kallikrein is pro-urokinase. Ichinose et al. [141] reported that the zymogen of this plasminogen activator can be converted by plasma kallikrein into a disulfide bond-linked two-chain form that degrades fibrin, its physiologic substrate; interestingly, these same Authors showed that thrombin converted pro-urokinase into a two-chain form that was not activatable by kallikrein or other serine proteases.

#### Mast cell serine proteases

Mast cells are mostly known for their role in allergic reactions during which vasoactive substances like histamine and cytokines as well as proteases are massively released [142]. Mast cells also play a role in the clearance of foreign bodies, including bacteria. Although cathepsin G can be expressed by mast cells, tryptase and chymase are the major serine proteases stored in their granules [7].

While mast cell-derived serine proteases have been shown to activate PAR_2 _[143] their proteolytic activity has been also shown to target specific proteins widely implicated in cardiovascular physiology and pathology. Tryptase has been shown to convert pro-urokinase into its active form [144], and chymase promotes the release of TGF-β, a pro-fibrotic growth factor and potent angiogenesis inducer, from the endothelial extracellular matrix [145]. Mast cell serine proteases have also a potential impact on vascular tone and blood coagulation. In fact, chymase has been shown to cause the *in vitro *conversion of angiotensin I into angiotensin II, a protein implicated in vasoconstriction and post-infarction remodeling of the heart [146]. Conversely, tryptase may impact blood coagulation by depleting fibrinogen via its proteolytic degradation [147].

### Other protein targets of serine proteases that affect the cardiovascular system

Besides thrombin proteolysis of HMW FGF-2 (see above), studies from the early 70's had revealed that the hormone-like action of trypsin, another serine protease, is due to its effect on the insulin receptor [148,149] which generates a truncated insulin alpha-subunit receptor with an intrinsic signaling activity [150]. Lafleur et al. [151] have also reported a mechanism of thrombin signaling in human endothelial cells which does not appear to involve PAR_1_, PAR_2_, or PAR_4_. These Authors demonstrated that thrombin efficiently cleaves the 64 kDa form of membrane-type 1 matrix metalloproteinase (MT1-MMP) in the presence of cells. Thrombin also rapidly increased cellular MT1-MMP expression and activity.

In the following paragraphs we will discuss two proteins whose implication in cardiovascular disease has been clearly established but for which the impact of their interaction with inflammatory cells and proteases on their activity in the cardiovascular system remains to be elucidated.

#### C-reactive protein (CRP)

CRP, an acute phase reactant, has been recognized in recent years as an important cardiovascular risk factor and independent predictor of cardiovascular events [1,152]. CRP is considered a protein actively involved in atherogenesis, probably via the amplification of the vascular inflammatory response. CRP modulates neutrophil function [153,154] and up-regulates TNF-α, IL-6, IL-1β [155], MCP-1 [156], and tissue factor production by monocytes [157,158] as well as low density lipoprotein (LDL)-uptake [159], probably contributing to foam cell formation in atherosclerotic plaques. Using a model of vein grafting into arterial circulation our group reported that CRP levels increased peaking 24 hours after surgery in serum as well as within the vein graft [160]. *In situ *hybridization and Northern blotting analysis, however, showed no CRP mRNA in either the arterialized vein graft (AVG) or control veins whereas a strong positive hybridization signal was detected in the liver. Early increases in AVG-associated CRP levels after arterialization are nonetheless indicative of inflammatory processes. Whether or not neutrophil release of serine proteases plays an active role in localizing circulating CRP to the vein graft remains to be determined.

It has been suggested that CRP may act as an atherothrombotic agent as it inhibits endothelial NO synthase [161] and prostacycline [162], induces expression of plasminogen activator inhibitor-1 [163], and promotes tissue factor activity in monocytes [157]. Accordingly, a recent study reported increased thrombosis after arterial injury in transgenic mice over-expressing human CRP [164].

#### Osteopontin

Osteopontin is an aspartic acid-rich, N-linked glycosylated secreted phosphoprotein also known as extracellular matrix cell adhesion protein. Osteopontin plays a pivotal role in cell adhesion, chemotaxis, prevention of apoptosis, invasion, and migration of various mesenchymal, epithelial, and inflammatory cells. Osteopontin is over-expressed in renal and cardiovascular cells during tissue remodeling and in inflammatory cells associated to different clinical conditions [165]. Scatena et al. [166] described osteopontin as a multifunctional molecule highly expressed in chronic inflammatory and autoimmune diseases, and thus believed to exacerbate inflammation linked to atherosclerosis [167]. Specifically, plasma osteopontin levels correlate with the presence and extent of coronary artery disease [168] and high levels of the protein have been found in patients with restenosis after percutaneous coronary intervention [169]. Furthermore, Golledge et al. [170] have shown that serum and tissue concentrations of osteopontin are associated with abdominal aortic aneurysm. These observations suggest that osteopontin may play a role in plaque formation and vascular disease progression. Several protease cleavage sites have been identified in the osteopontin molecule that may be important in regulating its activity [171]. Osteopontin was characterized as a substrate for several MMPs [172] but it also contains a domain that requires cleavage by the serine protease thrombin to be functional [173]. A functional serine-valine-valine-tyrosine-glutamate-leucine-arginine (SVVYGLR) domain has a cryptic structure in intact osteopontin and its cleavage by thrombin or matrix metallo-proteases is required in order to be functional [173]. Osteopontin structure/function studies mapped its activities to the SVVYGLR heptapeptide motif in the thrombin-liberated N-terminal domain (SLAYGLR in mouse osteopontin). *In vitro *studies showed that the SVVYGLR cryptic domain exposed after thrombin cleavage is able to induce adhesion and migration [174], which highlights its importance in the inflammatory process. Osteopontin levels were found significantly elevated in rheumatoid arthritis patients and appeared to correlate with the serum levels of inflammation markers [175]. An *in vivo *study in a mouse model of rheumatoid arthritis using a neutralizing antibody directed specifically to the SLAYGLR domain (within the osteopontin N-terminal domain in rodents) showed that adding the synthetic heptapeptide SVVYGLR sequence greatly reduced proliferation of normal synovial cells leading to bone erosion and infiltration of inflammatory cells. The SVVYGLR peptide has also been shown to induce angiogenesis both *in vitro *and *in vivo *[176]. Giachelli et al. [177] elucidated the role of osteopontin in inflammation and reported that neutralizing antibodies to osteopontin blocked macrophage infiltration. Later, this group showed that neutralizing osteopontin reduced neointima formation [165] and Lai et al. [178] demonstrated in a mouse model that the osteopontin peptide SVVYGLR activates vascular pro-MMP9 and superoxide signaling. These data demonstrate the potential role of the osteopontin SVVYGLR sequence in signaling, and the importance of the regulatory mechanisms that control inflammatory diseases as well as the potential benefit in selective inhibition of osteopontin SVVYGLR signaling as a strategy to reduce inflammatory vascular remodeling [179].

The potential role of osteopontin over-expression or deficiency in atherosclerotic lesion formation has also been explored. Osteopontin is highly expressed in atherosclerotic lesions, especially in association with macrophages and foam cells [167,180,181]. On the other hand, Matsui et al. [182] generated osteopontin-null mice, crossed them with apolipoprotein (apo) E-deficient mice and found that female mice with osteopontin^+/-^/apoE^-/- ^and osteopontin^-/-^/apoE^-/- ^genotype had significantly smaller atherosclerotic and inflammatory lesions as compared to osteopontin^+/+^/apoE^-/- ^mice. They also found that the vascular areas with deposition of minerals in 60-week-old male osteopontin^-/-^/apoE^-/- ^mice were significantly increased as compared to those observed in osteopontin^+/+^/apoE^-/- ^mice. This report was consistent with findings that mice deficient in both matrix Gla protein, a vitamin K-dependent calcium-binding protein that plays a role in calcification balance, and osteopontin had increased calcification in their arteries compared to mice that were wild-type for osteopontin and homozygous for matrix Gla protein deficiency, suggesting an inhibitory effect of osteopontin on vascular calcification [183]. However, osteopontin circulating levels correlate with both mitral valve disease secondary to rheumatic fever [184] and the presence and calcification levels of stenotic aortic valves [185]. These data further highlight the role of osteopontin in inflammation, but also suggest that in spite of relatively high concentrations in circulation, osteopontin biological activity might be controlled by its post-translational modifications. Proteolysis of osteopontin could therefore play a key role in controlling the biological role of this multifunctional protein in the context of heart valve disease [167,186,187] as well as of other pathologies.

## Conclusions

**Figure 3:**
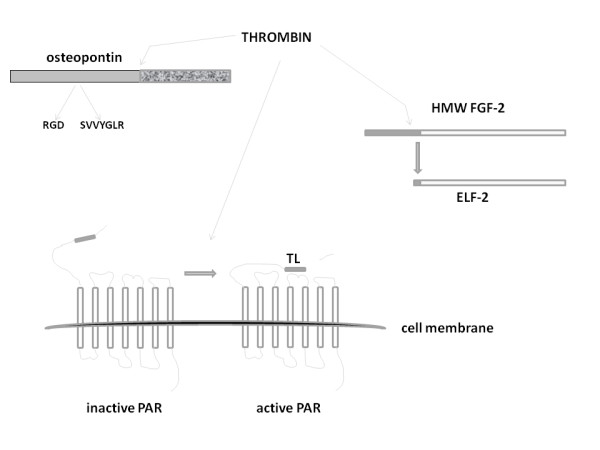
**Thrombin can act through PAR activation and/or by targeting specific protein substrates present in the vasculature such as osteopontin and HMW FGF-2**. PAR activation occurs when thrombin cleaves the seven transmembrane GPCR generating a tethered ligand (TL, small gray bar) that triggers a cascade of intracellular signaling events. Thrombin can also cleave a number of alternative substrates. Osteopontin cleavage exposes its functional domains: integrin-binding sites with RGD and SVVYGLR domains (grey bar) plus the C-terminal CD44-binding domain (marbled grey bar) critical for cellular recognition/interaction. HMW FGF-2 cleavage by thrombin leads to generation of vasoactive ELF-2 (see text and Figs. 1 and 2). Cleavage of either protein results in modifications of their biological activity which may have profound repercussions on cardiovascular homeostasis. Elucidation of these mechanisms may lead to specifically inhibit and/or control thrombin activity on specific protein substrates without affecting its coagulation properties.

The inflammatory response to tissue injury is characterized by recruitment and activation of immune cells with localized release of reactive oxygen species, serine proteases, and activation of the enzymes of the coagulation cascade. Such response although pivotal for the organism's defense may also lead to secondary effects not always beneficial to the homeostasis of the vascular bed and of the injured tissue. This might be particularly true when inflammation evolves from an acute response to a chronic condition. As a result, a number of biochemical modifications of the extracellular environment due to discharge of reactive oxygen species and activation of proteolytic enzymes in turn affects a number of molecular targets by either activating them or by suppressing their biological activity. Here we have focused on serine proteases involved in this process and their role in modifying proteins that are relevant to cardiovascular disease. The discovery over two decades ago that serine proteases such as thrombin can affect the vascular cell phenotype by activating specific cell membrane receptors, PARs, with a consequent cascade of intracellular signaling events shed a new light on our understanding of their mechanism of action. Nonetheless, serine proteases also target key players of the vascular homeostasis including growth factors implicated in angiogenesis and intimal hyperplasia such as FGFs, EGFs, PDGFs and VEGFs, and inflammatory mediators that impact vascular and tissue remodeling such as chemokines, CRP, and osteopontin. It is therefore reasonable to conclude that serine proteases affect the cardiovascular system by both PAR-dependent and -independent mechanisms. Thrombin cleavage of the HMW forms of FGF-2 is a paradigmatic example of post-translational control of a growth factor which may have profound repercussions on tissue and vascular remodeling secondary to injury. The elucidation of these mechanisms bears a potentially significant impact on the clinical approach targeting the activity of serine proteinases; for example, specific tools affecting certain biological effects of thrombin without altering its involvement in the coagulation cascade could be of paramount importance in the peri-operative care of cardiovascular interventions (Fig. 3). The lessons learnt by the use of wide spectrum serine protease inhibitors such as aprotinin in cardiovascular practice represent a sort of cautionary tale as a more refined targeting of serine proteases should be preferred over a generalized suppression of protease activity whose increased effectiveness on bleeding and/or inflammation might come with a series of dreadful and fateful side effects for the patient [188]. In this light, an increased knowledge of the mechanisms of action of the various serine proteases holds the key to finely targeted therapeutic tools.

Tissue remodeling secondary to injury represents a major mechanism that alters vascular homeostasis and proteases, particularly those associated with activated leukocytes and with the coagulation cascade, play a key role in this process. Understanding their biology and their effects on specific targets will afford a comprehensive approach to control their activity and a brighter outcome to prevent and/or cure the pathologies associated to their unwanted effects.

## Competing interests

The authors declare that they have no competing interests.

## Authors' contributions

RS contributed to the writing of this manuscript. PJY contributed to the writing of this manuscript. JP contributed to the writing of this manuscript. ACG contributed to the writing of this manuscript. PM contributed to the writing of this manuscript. GP contributed to the writing of this manuscript. All authors read and approved the final manuscript.
